# Clay impact on reservoir quality in the Nubia Formation of Saqqara field, Gulf of Suez, Egypt

**DOI:** 10.1038/s41598-025-07801-0

**Published:** 2025-07-24

**Authors:** Abdelbaset M. Abudeif, Nasir Alarifi, Fathy Abdalla, Stefano Bellucci, Faten A. Tawfik

**Affiliations:** 1 Geology Department, Faculty of Science, Sohag University, Sohag, Egypt; 2 Geology and Geophysics Department, College of Science, King Saud University, Riyadh, Saudi Arabia; 3 Faculty of Science, Geology Department, South Valley University, Qena, Egypt; 4 Laboratory of Optical Processes in Nanostructured Materials, National Institute of Materials Physics, P.O. Box MG-7, Bucharest, R077125 Romania

**Keywords:** Shale volume, Reservoir quality, Porosity, Permeability, Clay minerals, Clay mineral’s structure, Shale distribution, Geophysics, Core processes

## Abstract

This study aims to investigate the impact of clays on the quality of the Nubia reservoir in the Saqqara field, Gulf of Suez, Egypt. The research will contribute to a broader knowledge of reservoir characterization, offering valuable insights for similar geological settings in other regions, thereby aiding in the optimization of resource management in global petroleum industries. The shale evaluation procedure involves three primary steps: estimating shale volume, identifying clay minerals, and assessing shale distribution. The neutron-density (N-D) method was employed to estimate the shale volume in the Nubia reservoir, yielding an average of 0.6% across four wells, with a maximum recorded value of 2.2% in well GS323-3. These values represent that the Nubia reservoir contains a negligible amount of shale, indicating that the porosity and permeability are high. Clay mineral analysis, based on a Potassium-Thorium (K-Th) and Potassium-PEF cross-plots, identified the presence of chlorite, illite, montmorillonite, and heavy thorium-bearing minerals, where chlorite and illite enhance the mechanical stability of the reservoir, while montmorillonite may cause issues with swelling and pressure. Thorium-bearing heavy minerals are typically associated with reduced permeability due to their influence on chemical interactions. The shale distribution analysis, conducted using the Thomas and Stieber model, confirms the overall cleanliness of the Nubia reservoir. Most formation data points align with the 0% shale line, indicating high total porosity, while only a few points fall along the dispersed shale line. In conclusion, the findings indicate that the Nubia reservoir exhibits minimal shale content, predominantly clean lithology, and favorable porosity and permeability characteristics. Consequently, the reservoir is classified as high-quality, making it suitable for efficient hydrocarbon production.

## Introduction

**Fig. 1:**
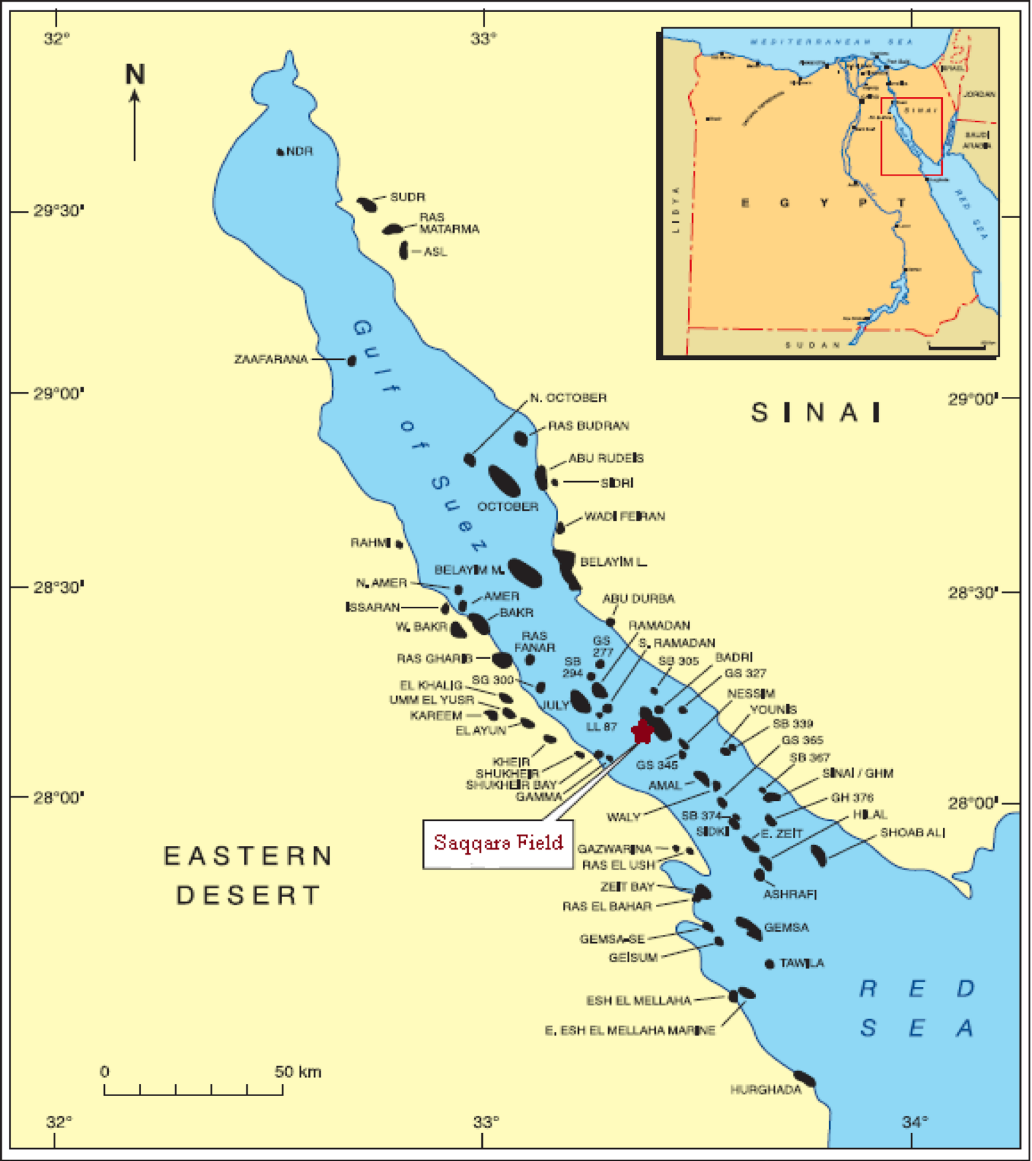
Location map of the Gulf of Suez including the location of the studied field, modified using CorelDraw^30^software after^31^.

The Gulf of Suez (GOS), as shown in Fig. 1, is a geologically significant region in Egypt, recognized for its complex tectonic structure and diverse sedimentary layers, making it a focal point for petroleum exploration and research^1^. Extending approximately 300 km in length and 30 to 40 km in width, the Gulf runs parallel to the Red Sea and has undergone extensive faulting and fracturing due to multiple tectonic events spanning from the Cretaceous to the Eocene periods. These geological processes have given rise to unique structural formations that are highly conducive to the accumulation of substantial hydrocarbon reserves, positioning the Gulf of Suez as one of Egypt’s most active and prolific petroleum-producing regions^1^.

**Fig. 2:**
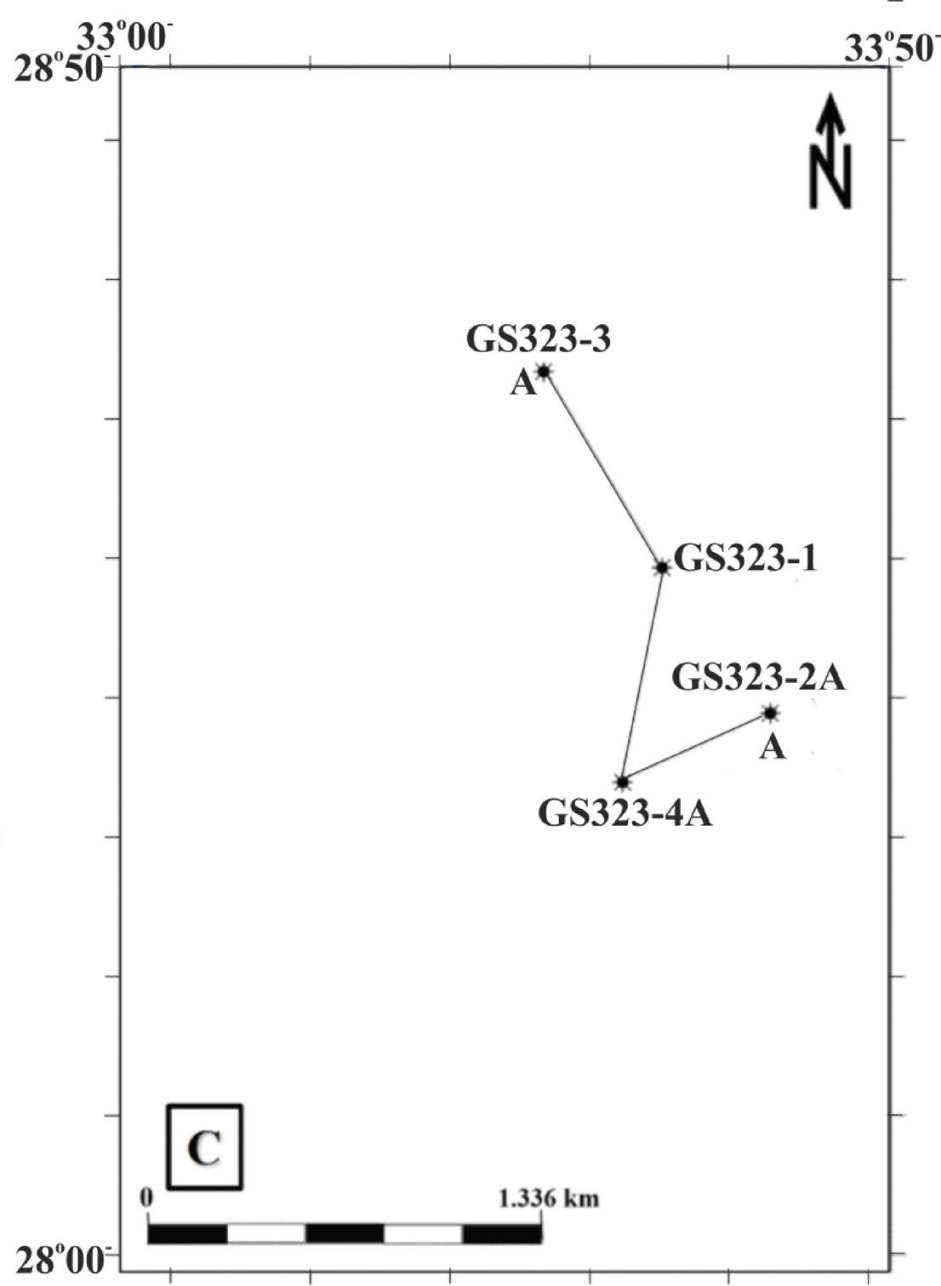
A base map showing the well locations in the Saqqara Field, including wells GS323-2 A, GS323-4 A, GS323-1, and GS323-3 ^15^.

The GOS is home to a variety of reservoir types, particularly notable for its Cretaceous sandstones and Eocene limestones. These reservoirs are characterized by a combination of low-porosity and porous rock types, which are ideal for both hydrocarbon migration and storage^2^. Among its largest fields is the Saqqara Field, located in the southern-central part of the Gulf. Discovered in 2003, the Saqqara Field continues to be an area of ongoing exploration. It is strategically situated 3.5 km east of the Edfu Oil Field, 7.5 km south of the Ramadan Oil Field, and 3.5 km west of the El Morgan Field. Spanning longitudes 33° to 33°50’ E and latitudes 28° to 28°50’ N^3^(Figs. 1 and 2), the Saqqara Field stands out due to its promising potential and hosts two primary reservoirs: the Matulla and Nubia reservoirs. These reservoirs are particularly noteworthy for their favorable petrophysical properties, including high porosity and permeability, which contribute to their efficiency as hydrocarbon storage units. The substantial sandstone and limestone deposits within these reservoirs enable significant oil and gas accumulation, enhancing their commercial viability^4^.

The global significance of this work lies in its contribution to understanding shale’s role in hydrocarbon reservoir characterization. This study provides a detailed evaluation of how shale volume influences porosity, permeability, and hydrocarbon storage in one of the world’s key petroleum-producing regions. The findings are critical for optimizing exploration and production strategies globally, especially in comparable rift basins, enhancing energy security, and advancing reservoir modeling techniques for sustainable hydrocarbon recovery.

To assess the impact of clay content on the quality of the Nubia reservoir, four wells (GS323-1, GS323-2 A, GS323-3, and GS323-4 A) were selected for detailed analysis. The volume of shale in a reservoir plays a critical role in determining its overall quality, as it directly influences both permeability and porosity. High shale content typically reduces permeability, as shale fills pore spaces, impeding fluid flow and hydrocarbon migration, which in turn diminishes productivity. Moreover, shale volume affects other key reservoir properties, such as stability, pressure dynamics, and fluid saturation, further influencing overall reservoir performance. Reservoirs with significant shale content may exhibit unpredictable property variations, complicating reservoir management and reducing hydrocarbon recovery efficiency. Therefore, understanding the relationship between shale volume and reservoir quality is essential for optimizing resource management and enhancing extraction strategies. This study aims to investigate how variations in shale content affect permeability, porosity, stability, pressure, and fluid saturation, with the goal of developing strategies to improve reservoir performance and maximize hydrocarbon recovery.

The Nubia reservoir has been the subject of extensive research, with numerous studies focusing on its geological, petrophysical, and reservoir characteristics. Notable contributions from a wide range of authors, spanning from 2012 to 2024, have provided valuable insights into the reservoir’s stratigraphy, hydrocarbon potential, and overall quality. These studies have explored aspects such as sedimentology, diagenesis, and tectonic influences, significantly advancing our understanding of the reservoir’s behavior and informing strategies for its efficient exploitation. The previous studies in this work include the following: (1) Geological Studies, which focus on stratigraphy, lithology, and regional geology ^5-10^ (2) Petrophysical Analysis, which examine petrophysical properties and log interpretation ^11-16^(3) Reservoir Evaluation, which research reservoir quality, potential, and productivity ^17-22^and (4) Tectonics and Structural Geology, which focus on structural analysis, fault systems, and the tectonic framework^8,23^. These works have made substantial contributions to the knowledge base, helping to refine resource evaluation methods and improve reservoir management techniques as ^5–14,17−20,22–29^.

**Fig. 3:**
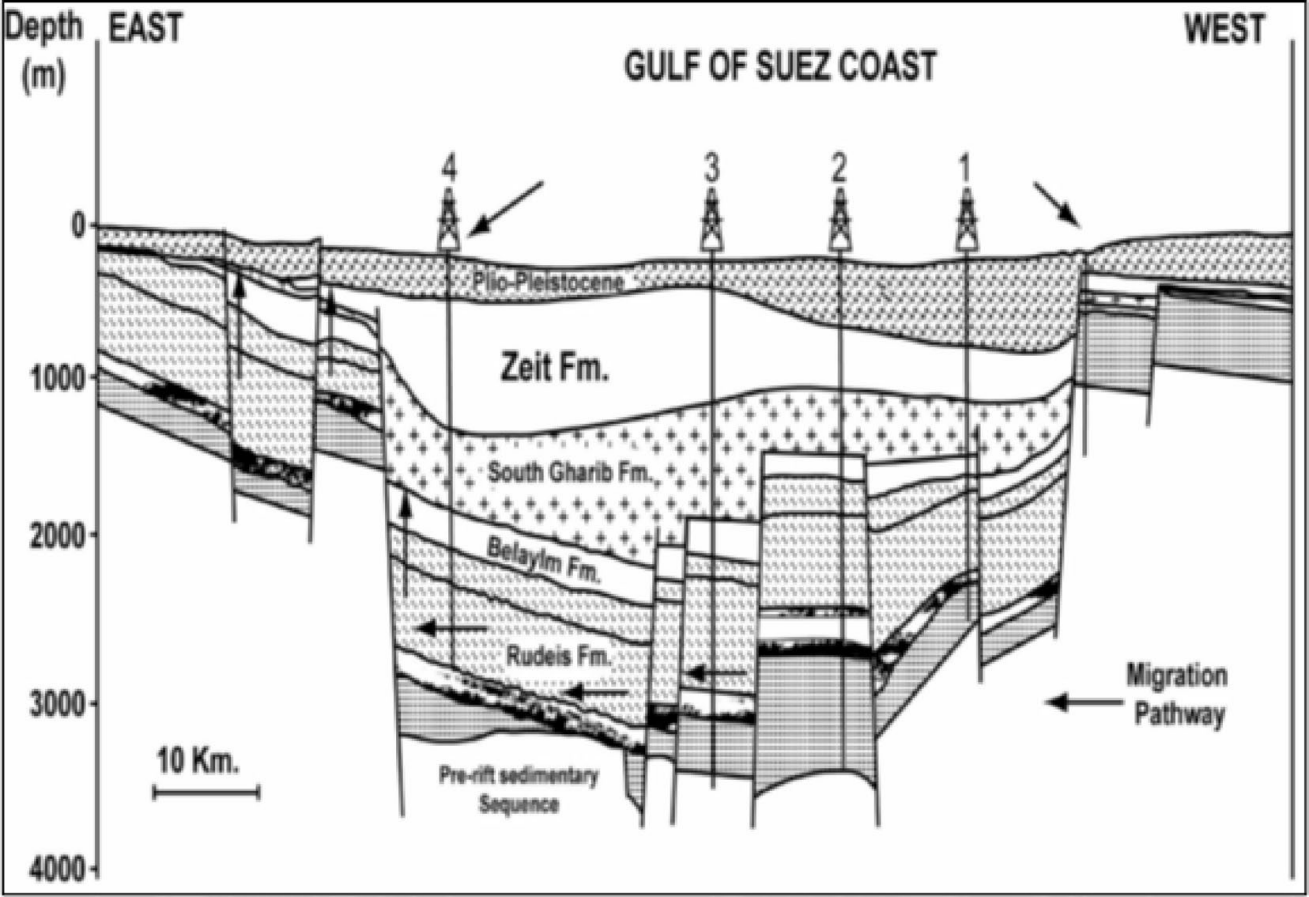
A cross-section illustrating the highly faulted structure of the Gulf of Suez^32^.

## Geologic setting

The Red Sea rift system, which became active between the Late Oligocene and the Miocene, is represented by the Gulf of Suez (GOS), which is its northern extension^33^. The GOS remained tectonically separate, shutting early during the rifting phase, whereas the Red Sea reached the stage of oceanic spreading ^34-37^. The Dead Sea Transform, a major plate boundary that caused tectonic activity to move eastward during this evolutionary phase, is primarily responsible for the current motion between the African and Arabian plates north of the Red Sea^34^.

During its brief post-rift phase, water flooded the lowest parts of the residual rift topography, forming the Gulf of Suez. It is among the most faulted areas on Earth, impacted by the movements of the Arabian, Sinai, and Nubian plates (Fig. 3). The E-W (Tethyan) and NNE-SSW (Aqaba) trends that resulted from these migrations have greatly influenced the Gulf’s present geological structure. These fracture networks may still be active now, adding to the tectonic complexity of the area^38^.

The tensional movement and subsequent subsidence caused crustal strain and collapse. This process facilitates the transport of potential source rocks to depths suitable for hydrocarbon generation within rift basins. Simultaneously, the stretching movement forms structural traps within fault blocks, which are ideal for accumulating hydrocarbons^39^.

**Fig. 4:**
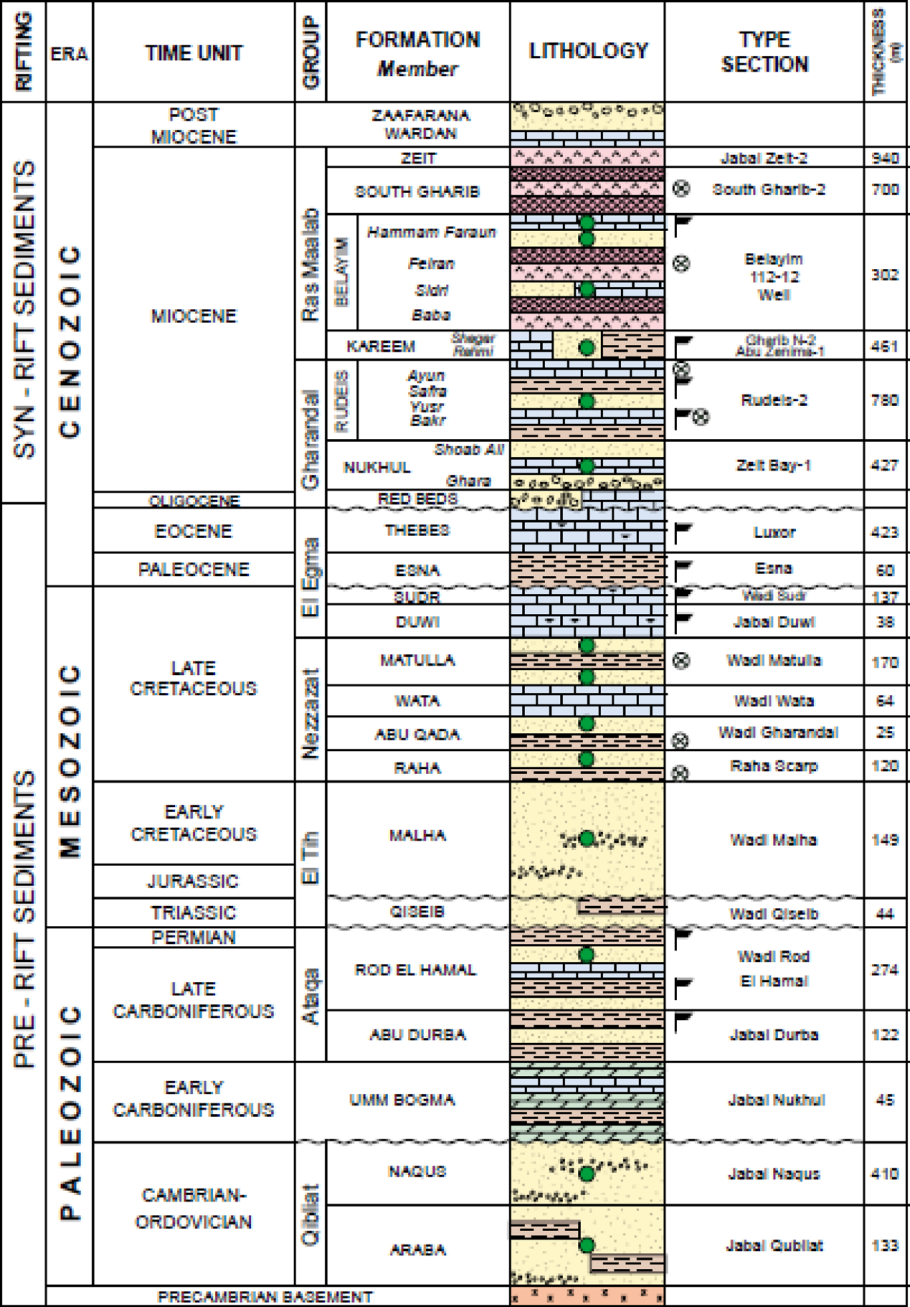
Simplified stratigraphic column showcasing the geological formations of the Gulf of Suez, highlighting the major lithological units and their chronological arrangement^1^.

The lithostratigraphic units in the Gulf of Suez range in age from the Pre-Cambrian to the Holocene. A simplified and generalized stratigraphic section representing the study area is depicted in Fig. 4. The stratigraphic succession of the Gulf of Suez, shaped by Cenozoic rifting, is categorized into three lithostratigraphic mega-sequences: pre-rift, syn-rift, and post-rift^40^. Each sequence exhibits distinct characteristics in terms of hydrocarbon potential, lithology, thickness, spatial distribution, and depositional environments^7^.

The pre-rift stratigraphic sequence extends from the Cambrian to the Eocene, comprising sediments deposited atop the tectonically stable Pre-Cambrian basement^41^. This sequence begins with clastic Nubian sandstones formed during the Paleozoic to the Lower Cretaceous Albian, followed by a marine transgression phase that influenced the deposition environment^42^.

A marine incursion resulted in the deposition of interbedded carbonate and clastic sequences. The Upper Cretaceous succession includes, in ascending order, the Raha, Abu Qada, Wata, Matulla, Brown Limestone, and Sudr (Chalk) formations. During the Paleocene and Eocene, shales, carbonates, and marls were deposited, reflecting the continuation of marine transgression from the Cenomanian. This transgressive regime culminated with the Eocene deposits, marking the final phase of this prolonged depositional environment^42^. The syn-rift stratigraphic series comprises Miocene and Oligocene sediments. During the Oligocene, the Gulf region was dominated by continental or shallow marine conditions^43,44^.

This study emphasizes the Nubia Formation of the Late Cretaceous, characterized by marls, limestones, shales, and multiple sandstone strata. The formation’s thickness typically ranges from 70 to 500 feet, reflecting its significant contribution to the geological and reservoir characteristics of the area.

The upper portion of the Matulla Formation is characterized by sandy shales that transition into pure shales toward the top. Overlying it is the Brown Limestone member of the Sudr Formation, consisting of dark brown, organic-rich, and radioactive limestones. This transition marks a sharp boundary, indicating a shift to a distinct depositional environment^41^.

## Methodology

This study examines how shale affects the quality of the Nubia reservoir. Shale volume (Vsh) refers to the fraction or percentage of shale content within the reservoir rock. It significantly influences reservoir quality by affecting both its capacity to store hydrocarbons and the ease of hydrocarbon flow through the rock. Understanding this relationship is critical for assessing and enhancing reservoir performance^45^.

The evaluation of shale involves three main steps: identifying the types of clay minerals present, calculating shale volume (Vsh), and analyzing the distribution of shale within the reservoir rock. These processes are fundamental for understanding the impact of shale on reservoir characteristics and overall performance.

### Clay minerals identifications

Among the four studied wells, only GS323-4 A had spectral gamma-ray log data available, making it the primary source for clay mineral identification. However, the geological similarity and close spatial proximity of the wells suggest that the clay distribution is relatively uniform across the reservoir. This assumption is supported by the consistent petrophysical properties observed in all wells. Additionally, shale volume calculations were performed across all wells, further validating the findings.

Shale significantly impacts hydrocarbon reservoir evaluation, emphasizing the need for precise identification. Clay minerals in shale influence the reservoir’s storage capacity and production efficiency. While the Archie Equation assumes formation water as the sole conductive element, the conductivity introduced by shale complicates porosity assessment. This often results in high total porosity (Φt) but reduced effective porosity (Φe), affecting the reservoir’s hydrocarbon potential. Understanding these dynamics is critical for evaluating shaly formations as viable hydrocarbon reservoirs ^46-48^. Spectral Gamma Ray (SGR) measurements, which include Thorium (Th), Potassium (K), and Uranium (U), along with the Photoelectric Log (PEF), are effective tools for identifying clay minerals. This is achieved through cross-plots such as Thorium versus Potassium (Th-K), PEF versus Potassium (PEF-K), and PEF versus the Thorium-to-Potassium ratio (Th/K). These plots help characterize the mineralogical composition and distribution of clay within the reservoir.^49-53^. In this study the clay minerals were identified using the potassium-thorium and the PEF-potassium cross-plots.

### Shale volume calculation

Shale volume (Vsh​) estimation can be performed using various methods, including linear, non-linear, neutron-density (N-D), and self-potential techniques, with the choice of method depending on available data and reservoir conditions. For the Nubia Formation, the N-D method was selected due to its superior accuracy in formations containing K-feldspar-rich sandstones. In such formations, linear and non-linear methods tend to overestimate shale content due to the influence of heavy thorium-bearing minerals. To validate this selection, Table 1 presents a comparative analysis of different shale estimation techniques, highlighting their error margins, suitability for K-feldspar-rich sandstones, and geological applicability. The shale volume is determined using Equ (1):

### Shale type determination

**Fig. 5:**
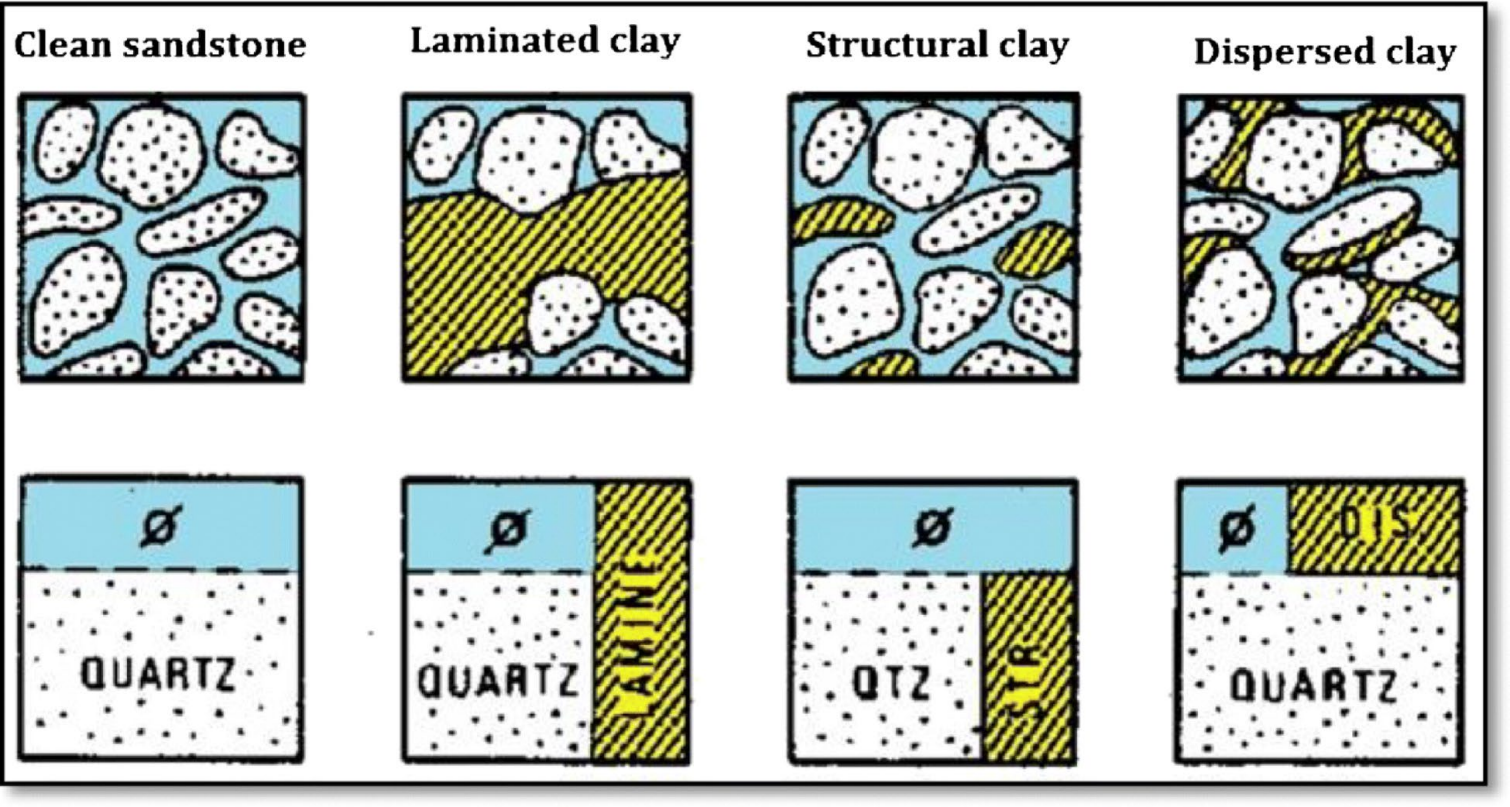
Schematic representation of shale distribution within rock pores, illustrating different clay arrangements: clean sandstone, laminated clay, structural clay, and dispersed clay. Modified after^62^.

The mineral composition of rocks is usually determined using microscopic techniques on thin sections. When physical samples are unavailable, wireline logs can be used as an alternative method for this analysis^16,54-56^. Three main shale types can be identified in sandstone formations (Fig. 5) : laminated shale, structural shale, and dispersed shale^57,58^.

#### Laminated shale

Laminated shale is a sedimentary rock that contains a high clay content, distinguished by thin, alternating layers of various clay types, which may differ in mineral composition, color, or texture. These layers usually develop in low-energy depositional environments, such as lakes or deep marine areas, where fine particles like clay and silt accumulate^59^. The laminar shale does not affect the porosity or the permeability of the sand streaks themselves, therefore, when the amount of laminar shale is increased the amount of porous medium correspondingly decreases and the overall average porosity is proportionally reduced.

**Table 1:** Comparison of shale volume estimation methods.

Method	Error margin (%)	Suitability for K-feldspar-rich sandstones	Advantages	Disadvantages	Equation used	Ref.
Neutron-density (N-D) method	Low (5–15%)	Good to excellent	More accurate in distinguishing shale from sand. More accurate in formations with high K-feldspar content	May be affected by clay minerals in the cementing material	$\:{V}_{sh}=\:\frac{({\varphi\:}_{N}-{\varphi\:}_{D})}{\left(\:{\varphi\:}_{N\:shale\:}-{\varphi\:}_{D\:shale}\right)}$ (1)	^52^
Self-potential (SP) method	Moderate to high (15–30%)	Poor to moderate	Useful for distinguishing permeable and impermeable formations	Sensitive to formation water salinity and requires calibration	$\:{V}_{sh}=\frac{{SP}_{Log}-{SP}_{Min}\:}{{SP}_{Max}\:-{SP}_{Min}\:}$ (2)	^60^
Linear methods	Moderate to high (10–25%)	Poor to moderate	Simple and easy to compute	Inaccurate in rocks with high feldspar content due to radioactive interference	$\:{V}_{sh}=\frac{{GR}_{Log}-{GR}_{Min}\:}{{GR}_{Max}\:-{GR}_{Min}\:}$ (3)	^47^
Non-linear method (Larionov’s for Old Rocks)	Low (5–10%)	Poor to moderate	More precise and consider complex rock formations	K-feldspar effects can inflate GR readings, leading to overestimation of shale volume	$\:{V}_{sh}=0.33\:*\left({2}^{2\:IGR}-1\right)$ (4)	^61^
Non-linear method (Larionov’s for Young Rocks)	Low (5–10%)	Poor to moderate	More accurate for younger formations	Similar to the previous model, but can overestimate shale in feldspar-rich formations	$\:{V}_{sh}=0.083\:*\left({2}^{3.7\:IGR}-1\right)$ (5)	^61^

Equation variables and definitions in Equ.(1 to 5)

(V_sh_) is the shale volume, ($\:\varphi\:$_N shale_) is the reading of the average neutron porosity in a pure shale zone, (ϕ_N_) is the reading of the neutron porosity for the formation, ($\:\varphi\:$_D shale_) is the average reading of the density porosity in a pure shale zone, and (ϕ_D_) ​is the reading of the density porosity for the formation, (GR_log_​) is the Gamma Ray log reading, (GR_min_​) is the GR value for clean sands, (GR_max_) is the GR value for shale, (SP_log_​) is the SP log reading, (SP_min_) is the SP value for clean sands, (SP_max_) is the SP value for shale, and (I_GR_) is the gamma ray index.

#### Structural shale

Shale can occur as grains or nodules within the formation matrix, and this shale is known as “structural shale”. It is generally thought to exhibit characteristics comparable to laminated shale and nearby the massive shale, in terms of its physical properties and behavior.

#### Dispersed shale

Shale materials can be scattered throughout the sand, particularly when they fill the intergranular voids. Dispersed shale may accumulate by attaching to or coating the sand grains, or it might partially occupy the smaller pore spaces. The presence of dispersed shale in these pores leads to a significant decrease in the formation’s permeability. These forms of shale can occur simultaneously in the same formation^62,63^.

**Fig. 6:**
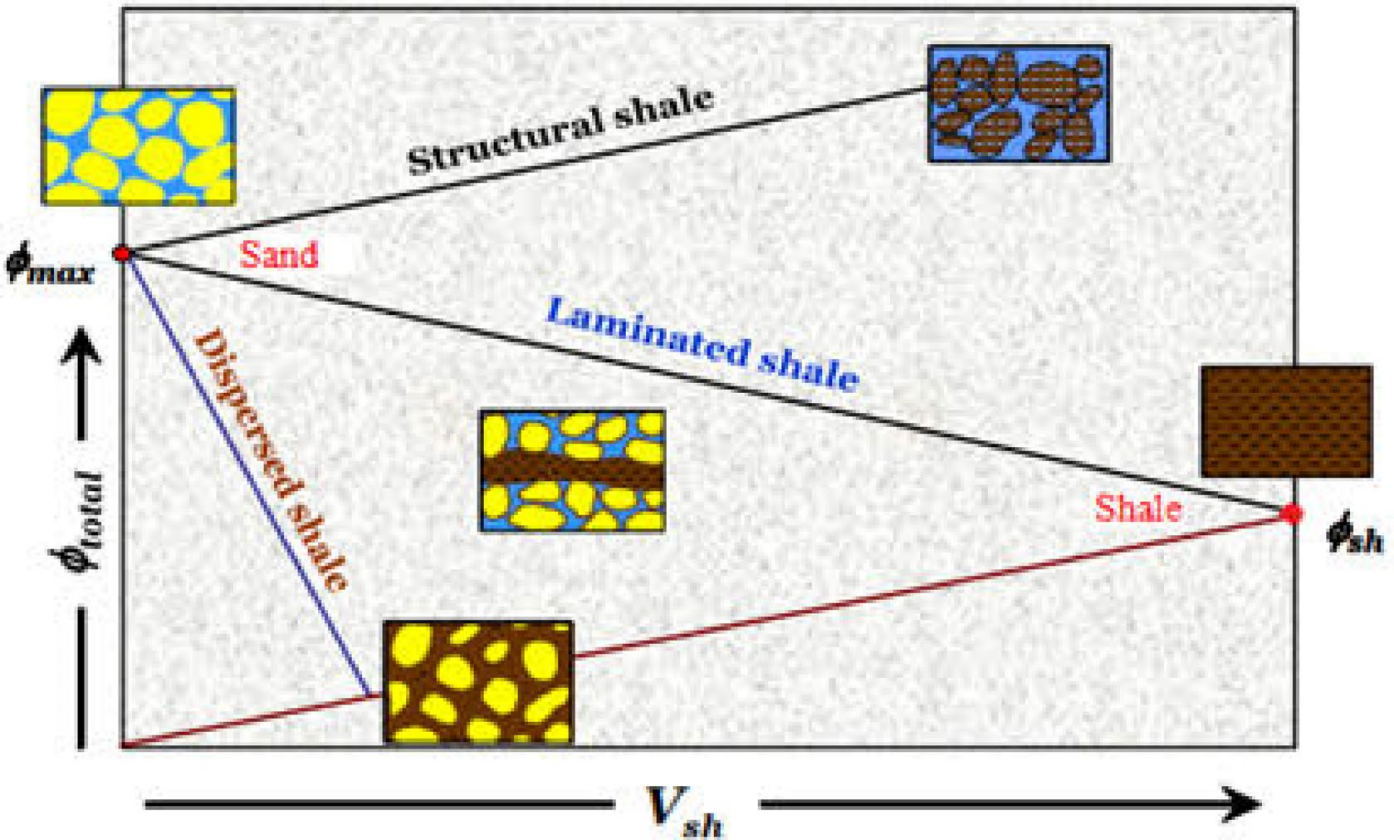
The Thomas and Stieber model used for shale distribution identification, illustrating different shale types (structural, laminated, and dispersed) and their impact on total porosity (ϕtotal​) as a function of shale volume (Vsh​). Modified after^63^.

The Thomas and Stieber model (Fig. 6) was applied on the Nubia reservoir to recognize the clay types^63^. This model consists of a cross-plot of shale volume (Vsh) versus total porosity (PHI_T).

## Results

### Clay mineral identification

**Fig. 7:**
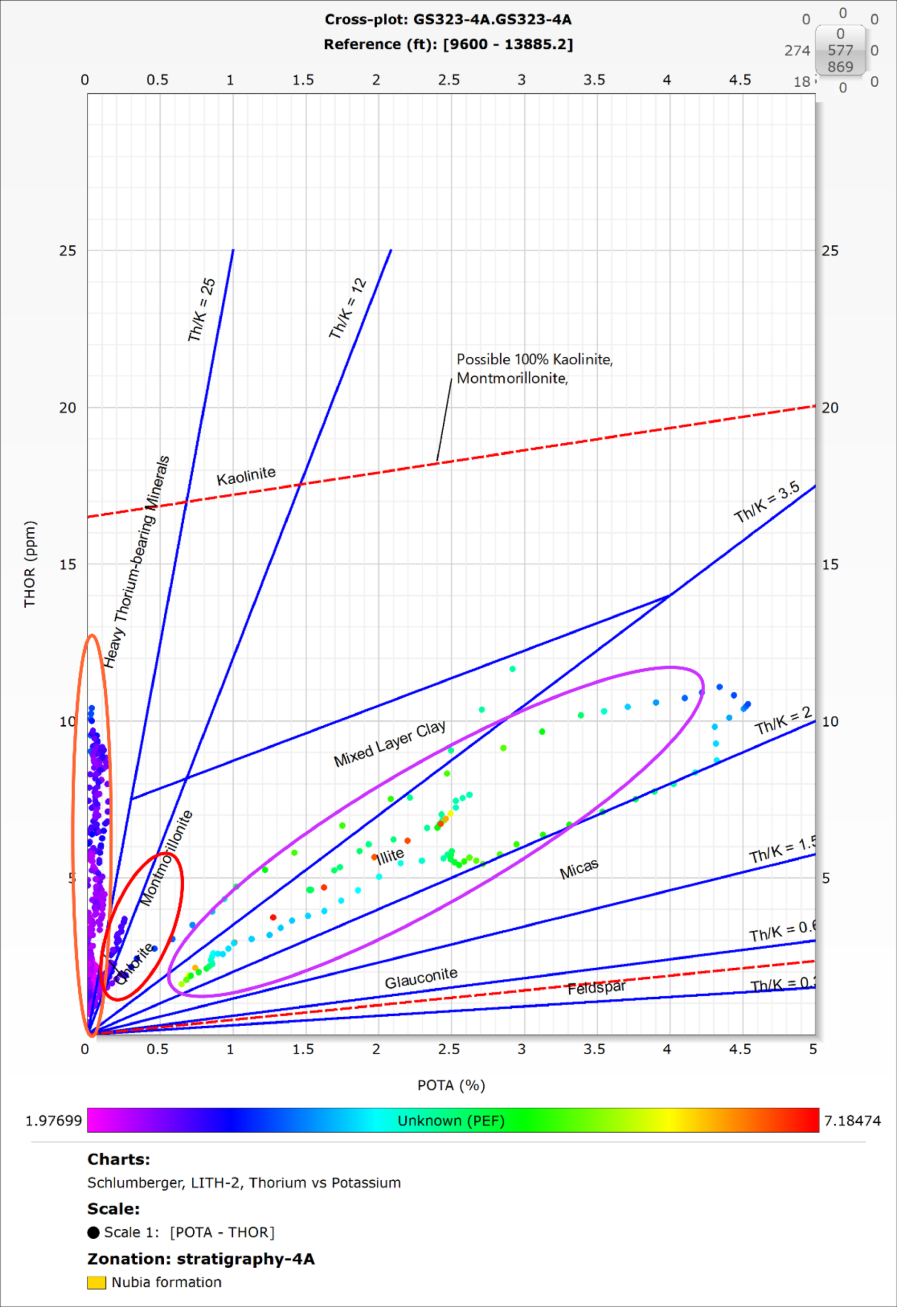
Thorium-potassium cross-plot of the Nubia reservoir in the GS323-4 A well, used for clay mineral identification. The cross-plot results indicate that the Nubia reservoir contains illite, montmorillonite, chlorite, and heavy thorium-bearing minerals, with the latter being dominant. These minerals significantly impact the reservoir’s porosity and permeability. The source of this figure is prepared using Techlog software^67^.

**Fig. 8:**
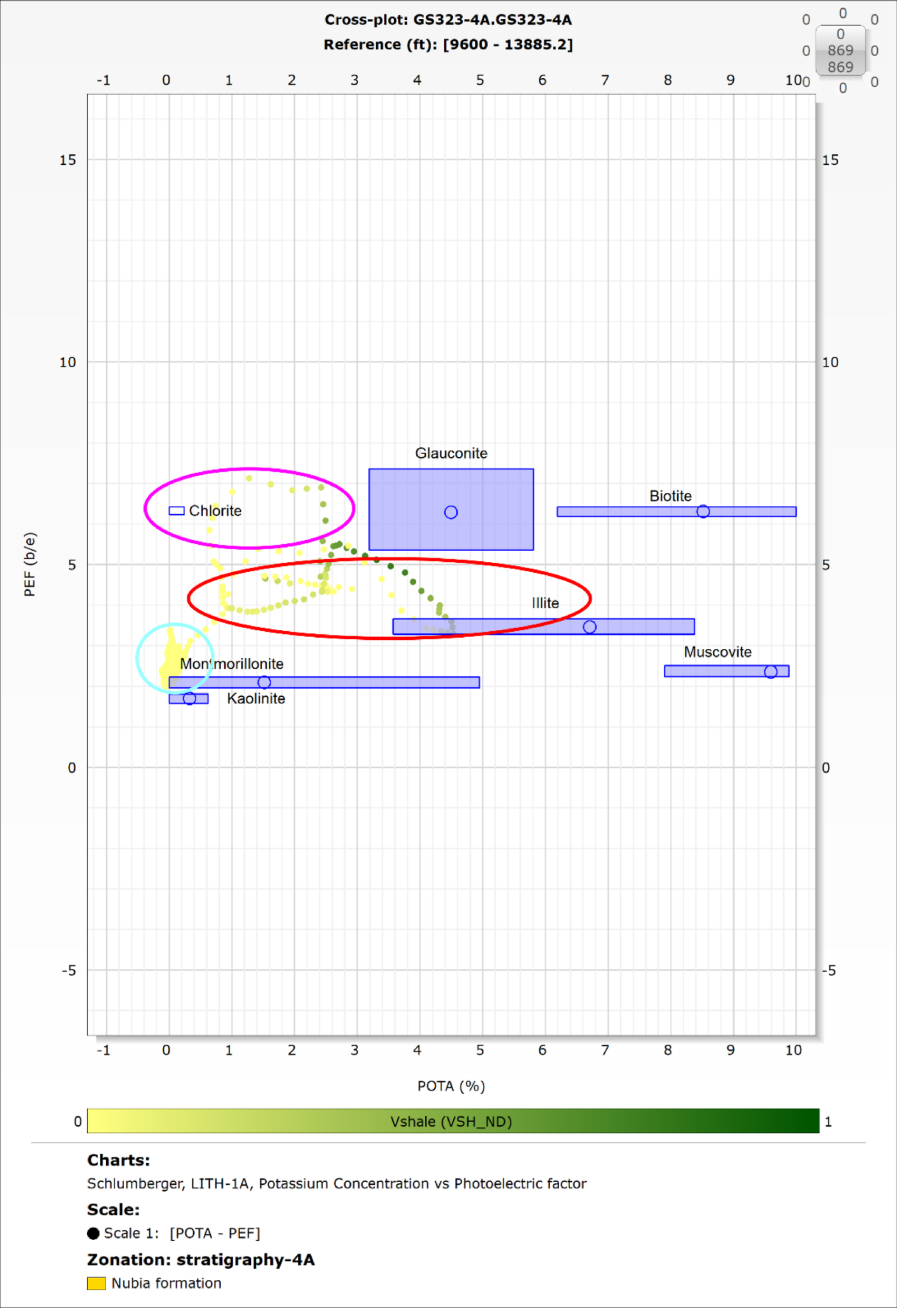
Potassium versus PEF cross-plot for the GS323-4 A well in the Nubia reservoir, highlighting the identification of clay minerals through analytical methods. The results reveal the presence of illite, montmorillonite, chlorite, and a dominance of heavy thorium-bearing minerals. These clay components notably influence the reservoir’s porosity and permeability characteristics. The source of this figure is prepared using Techlog software^67^.

The spectral gamma-ray log is provided only for the GS323-4 A well, therefore the potassium-thorium (Fig. 7) and the potassium- PEF (Fig. 8) cross-plots were exclusively applied to this well.

The results for both cross-plots indicate that the clay minerals in the Nubia reservoir include illite, montmorillonite, chlorite, and heavy thorium-bearing minerals. Heavy thorium- minerals are the predominant clay minerals, which significantly influences the reservoir’s porosity and permeability.

### The shale volume calculations

**Fig. 9:**
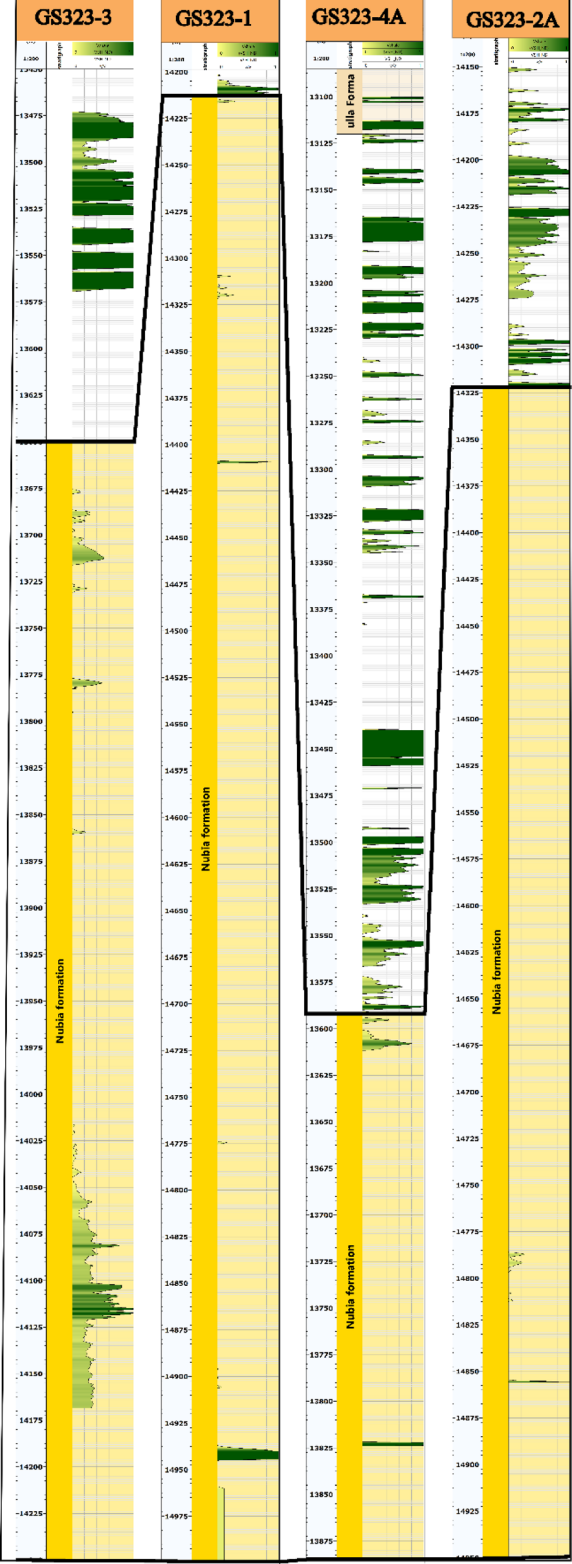
Cross-section (**A**-**B**) illustrating shale volume distribution within the Nubia Formation, connecting GS323-3, GS323-1, GS323-4 A, and GS323-2 A wells The source of this figure is prepared using Techlog software^67^.

Based on the application of the N-D method to the Nubia reservoir, the shale volume (V_sh_) values for the wells GS323-1, GS323-2 A, GS323-3, and GS323-4 A are very low, with average values of 0.1%, 0.1%, 2.2%, and 0% respectively. the maximum values reach 0.1% in some intervals within the studied reservoir (Fig. 9). The calculated shale volume values (0–2.2%) confirm that the Nubia reservoir is a clean sandstone formation with minimal shale content. Similar findings have been reported in other Nubian sandstone reservoirs within the Gulf of Suez^64–66^, supporting the validity of these results. The presence of minor shale content is attributed to dispersed clay minerals acting as cementing material rather than substantial shale beds.

### Shale distribution

**Fig. 10:**
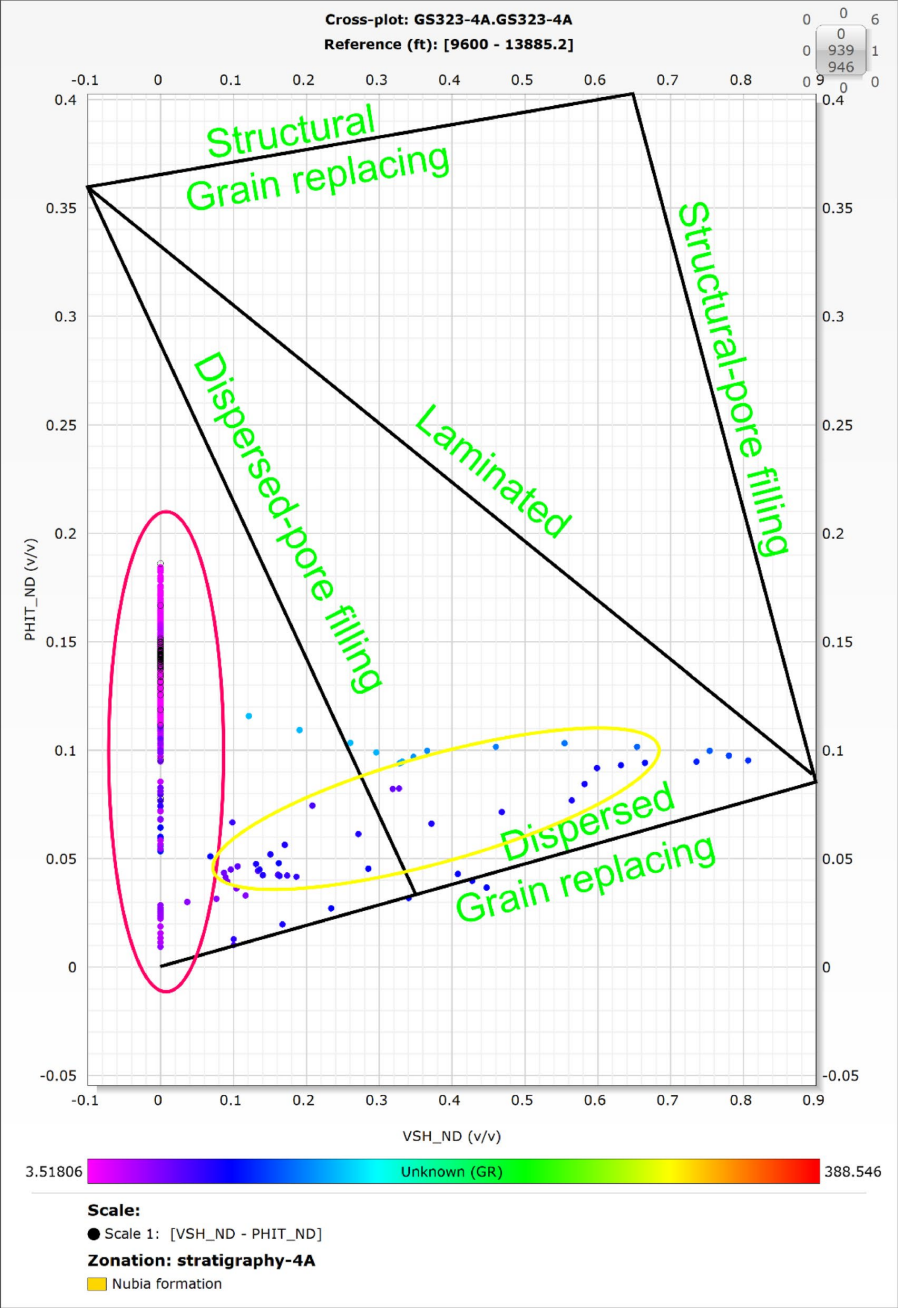
Thomas and Stieber model for the Nubia reservoir in GS323-4 A well. Most data points align with zero shale volume and high total porosity, indicating a clean reservoir, while fewer points reflect variable shale content. The source of this figure is prepared using Techlog software^67^.

The distribution of clay minerals within the Nubia reservoir is depicted in the cross-plot (Fig. 10) based on the Thomas and Steiber model. Most reservoir data points cluster around zero shale volume (Vsh) with high total porosity, indicating a predominantly clean reservoir. A smaller number of points are distributed along the dispersed trend line, reflecting zones with varying shale content.

## Discussion

### Clay minerals identifications:

Clay minerals play a crucial role in assessing reservoir quality, as they significantly impact porosity, permeability, and overall productivity. Their effects depend on the specific mineral type and its behavior within the reservoir.

The Nubia Formation contains heavy thorium-bearing minerals, chlorite, illite, and montmorillonite, primarily as cementing materials within the pore spaces. Among these, heavy thorium-bearing minerals have the most pronounced impact on reservoir quality.

#### Heavy Thorium-bearing minerals

Heavy thorium-bearing minerals are naturally occurring substances enriched with thorium (Th), a radioactive element commonly found in trace amounts in granitic rocks, pegmatites, and metamorphic environments. Important thorium-bearing minerals include monazite, thorianite, cuprosklodowskite, thorite, allanite, euxenite, zircon, and brannerite.

The presence of these minerals affects petrophysical properties, particularly porosity, permeability, and hydrocarbon storage capacity. If present in fine-grained form and significant amounts, they can reduce effective porosity and permeability by filling pore spaces and fractures, impeding fluid flow. However, in some cases, fluid-rock interactions or weathering can lead to mineral dissolution, potentially forming secondary porosity and improving permeability. Nonetheless, this is less common and highly dependent on reservoir conditions.

#### Illite and chlorite

Both illite and chlorite negatively affect porosity and permeability by forming fine fibrous structures that cover or fill pore spaces. Illite, due to its fine-grained nature, can cause pore throat blockage during production, further restricting fluid flow. Chlorite has a less severe impact than illite or smectite and, in some cases, may act as a protective coating, preventing the breakdown of other minerals. Additionally, both minerals enhance mechanical stability, reducing rock compaction risks.

#### Montmorillonite

Montmorillonite, a member of the smectite clay group, plays a critical role in reservoir quality due to its swelling behavior when exposed to water. Although present in low quantities within the Nubia reservoir, its ability to absorb water and expand significantly reduces permeability by constricting pore throats, hampering fluid flow, and increasing recovery difficulties. This swelling effect is particularly problematic during water-based drilling or injection processes, as it can cause pore blockage and near-wellbore formation damage, ultimately limiting hydrocarbon flow and reducing productivity.

### The shale volume calculations

Shale significantly impacts reservoir quality by reducing effective porosity while total porosity may remain relatively high. This occurs because clay within the shale retains water and occupies pore spaces, rendering much of the porosity less useful. Additionally, shale’s affinity for water increases irreducible water saturation, further reducing the space available for hydrocarbons. The presence of shales also negatively affects permeability, as shale particles obstruct pore throats, disrupt the connectivity between pores, and drastically limit fluid flow within the reservoir.

The average shale volume in the Nubia formation ranges from 0 to 2.5%, which means that almost there is a negligible amount of shale volume, and this very low percentage of shale is because of the clay minerals in the cementation materials of the Nubia sandstones, even so, it means that porosity and permeability are high, allowing fluids to move freely. This type of reservoir is generally considered high-quality and suitable for production.

Although the overall clay volume is relatively low, even minor clay content can exert a disproportionate influence on key reservoir properties—particularly in tight or low-porosity formations. In our study, we distinguish between total clay volume and the specific types of clay minerals present (e.g., kaolinite, montmorillonite, illite, and mixed-layer clays), as well as their spatial distribution within the pore network. Even in trace amounts, clay minerals can markedly affect reservoir characteristics such as porosity, permeability, and wettability. For example, pore-lining or pore-filling clays can significantly reduce effective porosity and impede fluid flow, thereby diminishing reservoir quality.

In unconventional reservoirs—especially those with complex mineralogical compositions and fine-grained textures—the specific clay mineralogy and its microstructural distribution may be more critical than total clay content alone. Our analysis demonstrates that these mineralogical features, though present in small quantities, can nonetheless have a substantial impact on hydrocarbon storage and production behavior.

To address this issue and clarify our perspective, we have revised the relevant sections of the manuscript to more clearly convey the significance of clay content in this context and have supported our discussion with additional literature^68-70^.

### Shale type determination

The Nubia reservoir demonstrates an almost negligible shale volume, as confirmed by the clustering of most data points at zero Vsh and high total porosity. This distribution supports the minimal impact of shale on the reservoir quality. However, scattered points along the dispersed trend line suggest the influence of clay mineral cementation, which may locally affect porosity and permeability within the formation.

Dispersed grain replacing shale, which has a limited effect because it occurs minimally, refers to a situation where fine-grained shale materials substitute the original rock grains, such as quartz or carbonate, throughout the formation. This replacement of grains by fine-grained shale may create a more consistent texture, but it can also reduce permeability due to the compacted nature of the shale matrix, which restricts fluid flow.

The integrated results indicate that the Nubia reservoir exhibits high quality, characterized by high porosity and good permeability, due to the extremely low shale volume (ranging from 0 to 2.5%). This low shale content suggests that the adverse effects of clay minerals on fluid flow are minimal. By combining the clay mineral identification through cross-plots (potassium-thorium and potassium-PEF) with the shale distribution analysis using the Thomas and Stieber model, it is evident that the localized influence of minerals such as illite, chlorite, and montmorillonite does not substantially compromise the overall petrophysical properties. Instead, these analyses reveal only minor variations that affect specific intervals within the reservoir. This integrated approach underscores that the reservoir quality largely depends on the minimal shale presence, which ensures open pore networks and effective fluid flow, thereby supporting the reservoir’s production potential.

## Conclusions

- This study focuses on the Saqqara Field, located in the south-central Gulf of Suez, to assess the impact of shale on the Nubia reservoir quality.
- The evaluation of shale influence was conducted in three key steps: estimating shale volume, identifying clay minerals, and analyzing shale distribution.
- Shale volume was estimated using the neutron-density (N-D) method, yielding values between 0% and 2.2%, indicating that the Nubia Formation contains negligible shale content.
- Clay minerals within the Nubia reservoir were identified through potassium-thorium and potassium-PEF cross-plot analyses, confirming the presence of heavy thorium-bearing minerals, montmorillonite, chlorite, and illite:
- Heavy thorium-bearing minerals can significantly reduce effective porosity and permeability by contributing to cementation and pore space filling, restricting fluid flow.
- Illite has a minor impact on permeability but remains relatively stable compared to other clays.
- Montmorillonite is highly reactive to water, swelling significantly and severely reducing permeability, which hinders hydrocarbon flow.
- Chlorite accumulates in pore spaces, reducing porosity, though its effect is less severe than that of montmorillonite.
- The shale distribution within the Nubia reservoir was analyzed using the Thomas and Stieber model, revealing that the formation contains minimal dispersed clays. While dispersed clays typically degrade reservoir quality, their limited presence in the Nubia Formation suggests a negligible impact on overall reservoir performance.

Overall, while the Nubia Formation is classified as a clean reservoir, this study highlights the role of dispersed clay minerals in influencing porosity and permeability. The limited presence of shale, primarily in the form of cementing material, has a negligible effect on reservoir quality. These findings enhance the understanding of shale and clay mineral distributions in similar sandstone reservoirs and provide valuable insights for hydrocarbon exploration and production.

## Data Availability

The data that support the findings of this study are available from Egyptian General Petroleum Corporation (EGPC) and the Gulf of Suez Petroleum Company (GUPCO), Egypt but restrictions apply to the availability of these data, which were used under license for the current study, and so are not publicly available. Data are however available from the corresponding author upon reasonable request and with permission of Egyptian General Petroleum Corporation (EGPC) and the Gulf of Suez Petroleum Company (GUPCO).

## Abbreviations

D: Density
GOS: Gulf of Suez
GR: Gamma ray
GR_log_: The gamma ray log reading
GR_max_: The GR value for shale
GR_min_: The GR value for clean sands
I_GR_: The gamma ray index
K: Potassium
N: Neutron
PEF: Photo electric factor
PHIE or Φeff: Effective porosity
PHIT or Φt: Total porosity
SGR: Spectral gamma ray
SP_log_: The SP log reading
SP_max_: The SP value for shale
SP_min_: The SP value for clean sands
Th: Thorium
U: Uranium
Vsh: Shale volume
Φ: Porosity
Φd sh: Density porosity of shale
Φd: Density porosity
Φn d: Neutron -density porosity
Φn sh: Neutron porosity of shale
Φn: Neutron porosity
